# Habitat configurations shape the trophic and energetic dynamics of reef fishes in a tropical–temperate transition zone: implications under a warming future

**DOI:** 10.1007/s00442-022-05278-6

**Published:** 2022-11-07

**Authors:** Nestor E. Bosch, Albert Pessarrodona, Karen Filbee-Dexter, Fernando Tuya, Yannick Mulders, Sahira Bell, Tim Langlois, Thomas Wernberg

**Affiliations:** 1 School of Biological Sciences, The UWA Oceans Institute, The University of Western Australia, 35 Stirling Highway, Crawley, WA 6009 Australia; 2 Institute of Marine Research, Nye Flødevigveien 20, 4817 His, Norway; 3 Department of Science and Environment, Roskilde University, 4000 Roskilde, Denmark; 4 Grupo en Biodiversidad y Conservación, IU-ECOAQUA, Universidad de Las Palmas de Gran Canaria, Crta. Taliarte S/N, 35214 Telde, Spain

**Keywords:** Community assembly, Ecosystem functions, Ecosystem services, Trait-based ecology, Tropicalisation

## Abstract

Understanding the extent to which species’ traits mediate patterns of community assembly is key to predict the effect of natural and anthropogenic disturbances on ecosystem functioning. Here, we apply a trait-based community assembly framework to understand how four different habitat configurations (kelp forests, *Sargassum* spp. beds, hard corals, and turfs) shape the trophic and energetic dynamics of reef fish assemblages in a tropical–temperate transition zone. Specifically, we tested (i) the degree of trait divergence and convergence in each habitat, (ii) which traits explained variation in species’ abundances, and (iii) differences in standing biomass (kg ha^−1^), secondary productivity (kg ha^−1^ day^−1^) and turnover (% day^−1^). Fish assemblages in coral and kelp habitats displayed greater evidence of trait convergence, while turf and *Sargassum* spp. habitats displayed a higher degree of trait divergence, a pattern that was mostly driven by traits related to resource use and thermal affinity. This filtering effect had an imprint on the trophic and energetic dynamics of reef fishes, with turf habitats supporting higher fish biomass and productivity. However, these gains were strongly dependent on trophic guild, with herbivores/detritivores disproportionately contributing to among-habitat differences. Despite these perceived overall gains, turnover was decoupled for fishes that act as conduit of energy to higher trophic levels (i.e. microinvertivores), with coral habitats displaying higher rates of fish biomass replenishment than turf despite their lower productivity. This has important implications for biodiversity conservation and fisheries management, questioning the long-term sustainability of ecological processes and fisheries yields in increasingly altered marine habitats.

**Supplementary Information:**

The online version contains supplementary material available at 10.1007/s00442-022-05278-6.

## Introduction

Understanding the role of deterministic and stochastic processes in community assembly is a central goal in ecology, particularly in the Anthropocene, where rapid changes to natural ecosystems challenge current management and conservation paradigms (Pecl et al. 2017; Bonebrake et al. 2018). Reef ecosystems are at the forefront of this biotic change, with recurrent coral bleaching events causing widespread coral mortality in tropical regions (Hughes et al. 2018; Dietzel et al. 2021). Likewise, gradual warming and extreme marine heatwaves, coupled with altered biotic interactions, are causing the collapse of kelp forests at the warm-edge of their range (Vergés et al. 2014, 2016; Wernberg et al. 2016). These foundational species provide the three-dimensional habitat structure that underpins their influence on biodiversity and associated ecosystem services such as food provision, coastal protection, and nutrient cycling (Jones et al. 1994; Romero et al. 2015). Across the globe, the replacement of these foundational species by structurally simple, opportunistic, fast-growing turf algae (Filbee-Dexter and Wernberg, 2018; Pessarrodona et al. 2021a, b, c) or alternative canopy-forming foundation species (e.g. *Sargassum* spp.) (Tanaka et al. 2012; Terazono et al. 2012) can fundamentally alter the structure of the associated faunal assemblages (e.g. fishes and invertebrates) (e.g. Stuart-Smith et al. 2018). However, their consequences for ecosystem functioning remains poorly resolved.

The ecosystem functions supported by novel habitat configurations is determined by the extent to which functional traits (i.e. morphological, physiological, or behavioural features) that increase the fitness of species in each habitat are filtered from the regional pool of species (Mcgill et al. 2006). Community assembly rules shape the occurrence and relative abundance of species coexisting locally, which is in part mediated by a trade-off between ecological constraints and opportunities provided by local abiotic (e.g. temperature, nutrients, primary productivity, wave exposure) (Bejarano et al. 2017; McLean et al. 2021; Bosch et al. 2021b) and biotic (e.g. habitat and resource availability, biotic interactions) conditions (Chase et al. 2009; Yeager et al. 2017). Proximal human pressures can also shape the number and identity of reef fish traits at local scales (D’agata et al. 2014), with extractive human activities (e.g. fishing) selectively impacting areas of the trait space (e.g. removal of large-bodied fishes) (Bosch et al. 2021a). Alternatively, local species coexistence can also be determined by stochastic processes (e.g. birth, death, immigration and emigration of individuals, i.e. ‘ecological drift’), irrespective of species’ functional identity (i.e. ‘neutral theory’) (Hubbell 2001). In reef fishes, this can be exacerbated by their generally large dispersal capacities, that enable them to colonize distant sites and compete for the available living space (Sale 1978). Although these community assembly processes are not necessarily mutually exclusive (Vellend et al. 2014; Bosch et al. 2021b), the prevalence of niche-based *vs.* neutral mechanisms is likely mediated by the arrangement of habitats across spatial scales (Yeager et al. 2011), and the degree to which species from the regional pool are habitat specialists or generalists (Stuart-Smith et al. 2021).

Effective management of transitioning reefs in the Anthropocene requires an understanding on how changes in the functional trait structure of ecological assemblages scales-up to shape ecosystem-level processes that underpin trophic and energetic dynamics (Bellwood et al. 2018; Vergés et al. 2019). This is paramount in the case of shifting habitat structure, as these changes can alter the composition of basal trophic levels that underpin the nutritional resources available to primary (Russ et al. 2015; Pessarrodona et al. 2021a, b, c) and secondary consumers (Taylor 1998; Fraser et al. 2020a, b; Fraser et al. 2021). Furthermore, evidence from coral reefs suggest that changes in ecosystem functions (e.g. productivity and turnover) that underpin the flow of energy and materials might remain undetected using static metrics such as species richness, abundance, and biomass (Morais and Bellwood 2019; Morais et al. 2020; Tebbett et al. 2021). Traditional approaches to estimate fish productivity rely on parameter estimation of trophodynamics (e.g. Christensen and Pauly 1992), limiting its use for high diversity systems for which these parameters are unknown for most species. The development of novel frameworks to estimate the productivity of reef fishes from survey and functional trait data have overcome this limitation (Morais and Bellwood 2020). However, these have rarely been quantified outside coral reef environments (but see Pessarrodona et al. 2021a, b, c), hindering generalizations about how habitat reconfigurations might impair the dynamic processes that sustain productive reef ecosystems.

Here, we took advantage of a tropical–temperate biogeographic transition zone characterized by the coexistence of a diverse array of habitats of varying structural complexity and thermal affinity. These habitats included reef configurations that have been predicted to arise in temperate reefs under future warming scenarios, as cool-affinity kelp forests (*Ecklonia radiata*) are potentially replaced by three biogenic habitat end-points: reef-building corals (*Acropora* spp.), mixed warm and cool *Sargassum* spp. beds, and turfs (Vergés et al. 2019). These habitat reorganizations are expected to shifts the ecosystem functions and services provided by temperate reefs, however, these have rarely been empirically quantified. In this study, we investigated (i) whether fish species coexisting in each habitat converge, or diverge, in their functional traits, (ii) which traits explain variation in species’ abundances across habitats, and (iii) how trait-based habitat filtering scales-up to shape energetic dynamics, measured via three metrics of energy storage and flow: standing biomass (kg ha^−1^), productivity (the amount of fish biomass produced per day, kg ha^−1^ day^−1^), and turnover (the proportional flow of energy in the system, as being incorporated or released, % day^−1^) (Morais et al. 2020). To elucidate how habitat reconfigurations might shift the trophic pathways that underpin fish productivity, we further partitioned these metrics for trophic guilds (herbivores/detritivores, microinvertivores, sessile invertivores, planktivores, and higher carnivores) that underpin trophic interactions and energy transfer in reef fishes (Parravicini et al. 2020).

## Materials and methods

### Study context

**Fig. 1:**
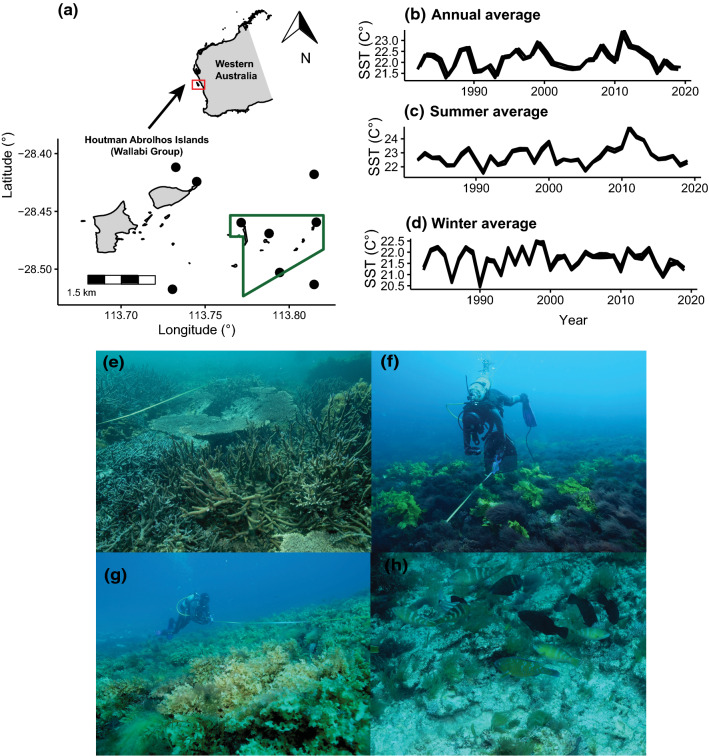
**a** Map of the study region (Houtman Abrolhos Islands, Wallaby Group, 28° latitude, ~ 60 km offshore) in the midwest bioregion of Western Australia. Black dots depict the spatial location of study sites. The green polygon delineates the no-take zone. **b**–**d** Yearly-averaged trends in **b** annual, **c** summer (December, January, February), and **d** winter (June, July, August) sea surface temperatures (SST, °C) from 1982 to 2020, across study sites. **e**–**j**. Photos of transect-level habitat clusters used in the analyses: **e** corals, **f** kelp, **g** *Sargassum* spp., and **h** turf

This study was conducted at the Wallabi Group of the Houtman Abrolhos Islands (28° 43′ S; 113° 47′ E), which lies ~ 60 km offshore in the tropical–temperate biogeographic transition zone of midwestern Australia (Fig. 1a). The islands lie within the main flow of the Leeuwin Current, a warm oligotrophic current that transports tropical propagules of marine biota into temperate latitudes (Pearce et al. 2011). The flow of the Leeuwin current is stronger in autumn and winter (Feng 2003), with average winter sea surface temperatures (SST) usually exceeding 20 °C (Fig. 1d), resulting in low intra-annual variability (~ 2–4 °C) (Fig. 1c, d). Since the 1980s, global ocean warming and sea-level rise has intensified the magnitude of warming anomalies (Zinke et al. 2014), whilst cooling phases have diminished in magnitude and frequency progressively (Fig. 1b–d), with profound changes in the marine flora and fauna of western Australia (Wernberg et al. 2016). The oceanography of the region, coupled with its southern location, facilitates a broad range of different habitats, including the southernmost true coral reefs in the Indian Ocean (Fairbridge 1950), temperate kelp forests and a mixed-pool of cool and warm *Sargassum* spp. beds (Phillips and Huisman 2009). Likewise, the associated invertebrate and fish assemblages comprises a diverse suite of warm- and cool-affinity species (Mulders et al. 2022).

### Fish and benthic surveys

Surveys were conducted in October 2020 (Austral spring) at nine sites distributed along a seaward (S-SW) to leeward (N-NE) orientation (Fig. 1a). Sites were a priori selected based on habitat maps for the study region (Evans et al. 2012) to capture the dominant benthic habitats that occur in shallow reef ecosystems: reef-building corals (*Acropora* spp.) (Fig. 1e), kelp (*Ecklonia radiata*) (Fig. 1f), *Sargassum* spp. (Fig. 1g), and turfs (Fig. 1h). All surveys were conducted during daylight hours (9 a.m. to 5 p.m.) on limestone shallow (5–12 m) reefs, which were separated by a minimum of 1 km. Sites were located both within and adjacent to a Fish Habitat Protection Area (i.e. no-take marine protected area for demersal and pelagic fishes, no-take zone herein) (Fig. 1a, Table S1). Due to the remoteness of the archipelago which entails low recreational fishing mortality (Bornt et al. 2015), and the relatively low contribution of targeted fishes to total abundance (3.78%) and biomass (26.57%) in the dataset, we expect no confounding effect of protection status on the patterns reported here.

We sampled fish assemblages using diver operated stereo-video surveys (stereo-DOV). Stereo-DOV is a robust technique to capture conspicuous fishes whilst minimizing inter-observer variability, with the stereo configuration allowing precise and accurate length measurements (Goetze et al. 2019). The stereo-DOV system consisted of two GoPro Hero 4 video cameras in underwater housings, mounted 0.7 m apart on an aluminium frame, converged at 8° to provide a standardized field of view. A full description of standard operating field procedures, configuration and calibration of cameras, is described in Goetze et al. (2019). At each site, eight replicated 25 × 5 m (125 m^2^) transects were swam by a SCUBA diver at a constant pace (~ 2 min per transect), with the cameras angled slightly downward, ca. 50 cm above the bottom, to enable the seafloor structure and composition to be observed. Replicated transects were spaced by at least 10 m to minimize non-independence of fish counts (Bosch et al. 2022; Mulders et al. 2022). Video transects were analysed in the ‘EventMeasure’ software (SeaGIS Pty Ltd), with all fishes within the transect area identified to the lowest taxonomic level possible, and their fork length measured (nearest mm). Individual lengths were converted to biomass using published length–weight relationships (Froese and Pauly 2021), with missing species assigned length–weight coefficients from a closely related species within the same geographic area.

The benthic composition and structure was quantified using a habitat annotation framework for forward facing imagery (Langlois et al. 2020). For each transect, we extracted *n* = 5 still images, evenly spaced along the transect (usually every ~ 20 s of video footage, ~ 4 m). Each frame was then divided in 5 × 4 grid cells of equal area (*n* = 20), and the dominant benthic biota scored within each grid. Benthic biota was classified in broad morpho-functional groups, following a classification scheme similar to CATAMI (Althaus et al. 2015): (i) coral (e.g. reef-building tabular and branching *Acropora* spp.), (ii) kelp (Laminarian canopy kelp, *Ecklonia radiata*), (iii) understorey erect red algae (e.g. *Callophycus* spp*.*), (iv) *Sargassum* spp. (complex-branching Fuculeans, mainly *Sargassum* spp.), (v) foliose (small, < 15 cm length, brown and red seaweeds; mainly *Lobophora* spp., *Padina* spp., *Zonaria* spp.), (vi) turf (single or multispecies aggregations of low-lying, < 2 cm, algae growing on bare rock or dead coral), (vii) cyanobacterial mats (dense aggregations of single or multispecies cyanobacteria), and (viii) sand (fine, unconsolidated, substratum). For each stereo-DOV transect, we computed the ‘percent cover’ of each morpho-functional group as the proportion of grid cells (%) each occurred, excluding grid cells where the benthos was not clearly visible (30% in total).

There was substantial heterogeneity in the percent cover of coral, kelp, *Sargassum* spp., and turf within the selected sites (Fig. S1a, b), resulting from the complex blend of habitat types that occur at the Abrolhos Islands over relatively small spatial scales (Evans et al. 2012). To minimize this confounding effect, we grouped transects that shared similar percent cover of habitat using a *k*-means clustering technique, implemented in the ‘fpc’ R package (Hennig 2010). The optimal number of habitat groups was estimated by iteratively fitting to an increasing number of clusters and comparing using the Duda-hart test. This resulted in six main habitat groups identified by the dominance of foliose algae (*n* = 9 transects), coral (*n* = 8 transects), kelp (shared dominance of canopy kelp *Ecklonia radiata* and understorey erect red algae, *n* = 15 transects), mixed (mixed blend of habitat types, *n* = 12 transects), *Sargassum* spp. (*n* = 20 transects), and turf (*n* = 8 transects) (Fig. S1b). For the purpose of the analyses, foliose and turf algae were grouped, as these habitats have been shown to share similar architectural properties that influence the composition of nutritional resources available to primary- and secondary-consumers, in comparison to coral, kelp, and *Sargassum* spp. habitats (Fraser et al. 2020a, b; Pessarrodona et al. 2021a, b, c). In addition, this grouping ensured a more balanced number of transects across habitat groups.

### Trait database

We compiled a database of species-level functional traits to capture ecological niche axes in reef fishes, potentially linked to their fitness in contrasting habitats (Mcgill et al. 2006). Traits selected represent key attributes of reef fishes, related to life history strategies, behaviour, trophic ecology and habitat utilization (Bosch et al. 2017): maximum length (numeric), gregariousness (ordered factor), water column position (factor), thermal affinity (numeric), and trophic guild (factor) (details are provided in Table S2). Trophic guild categories were assigned based on the dataset provided by Parravicini et al. (2020), which used network analyses based on quantitative gut content data and Bayesian phylogenetic modelling to predict the trophic identity of coral reef fishes. Species missing in the dataset, including those of temperate affinity, were assigned the trophic guild of a closely related species with similar body size and known behaviour, as these factors can accurately predict species’ trophic guild (Parravicini et al. 2020). Herbivores were further partitioned based on phenotypic traits that determine how they access the nutritional resources (e.g. algae, detritus, and cyanobacteria) contained in canopy- and turf-driven pathways (Bellwood et al. 2018; Siqueira et al. 2019; Bosch et al. 2022).

### Patterns of community assembly

We investigated the degree to which species co-occurring in each habitat type were functionally more similar (i.e. convergence, under-dispersed) or dissimilar (divergence, over-dispersed) than would be expected under random assembly mechanisms (Hubbell 2001). This was done by computing the functional diversity (FD) of each transect (α) (see raw values for taxonomic and functional diversity in each habitat in Table S3), and comparing the observed diversity to that expected under a null model. The FD was quantified using the attribute diversity framework (Chao et al. 2019), which measures the number of distinct functional entities’ in the sample, defined by a threshold level (*τ*) of trait dissimilarity (Chao et al. 2019); here, the average (0.35) dissimilarities in a Gower distance matrix. Hence, species below that threshold value belong to the same functional entity, whereas species above are part of distinct functional entities This framework enabled us to investigate patterns of community assembly, while varying the sensitivity of the metrics to the relative abundances of functional entities’, which are controlled by a parameter “*q*”: “*q*” = 0 (i.e., compositional changes only; occurrence-based; analogous to functional richness), “*q*” = 1 (i.e., higher weight on common entities; analogous to functional divergence), and “*q*” = 2 (i.e., higher weight on dominant entities, analogous to functional divergence). This enabled us to determine whether high stochasticity in species abundances overrides deterministic factors driving community assembly (Sale 1978). To test the sensitivity of the allocation of species into functional entities to the number of traits selected, we computed the correlation between the Gower distance matrix using all (5) traits and the Gower distance matrices after dropping one trait, at each time, from the analysis (4 out of 5 traits). In all cases, the correlation was high (> 0.87), suggesting trait selection would have no influence on the delineation of functional entities (Table S4) and, therefore, the robustness of our approach.

The null model was constructed by randomly reshuffling species identities from the regional pool of species, while maintaining species occurrence frequency and sample species richness. A standardized effect size (SES FD) was computed by iterating this procedure 999 times, and estimating the divergence between observed and expected diversity: SES = (obs–mean (null))/sd (null). We classified assemblages that were within the interquartile range of the null distribution as random, whilst assemblages below, or above, the 25th and 75th percentiles were considered to be predominantly under-dispersed and over-dispersed, respectively (Bosch et al. 2021b). Assemblages that differed significantly (> 95th percentile) were considered to be purely structured through deterministic assembly rules (i.e. abiotic and biotic processes). Analyses were carried out in R, via the ‘mFD’ package for computing FD (Magneville et al. 2021), and the ‘picante’ package for constructing the null models (Kembel et al. 2010).

### Fish trait–habitat relationships

We tested whether variation in fish species’ abundances across habitats was mediated by their functional traits using two complementary approaches. First, we used a model-based approach to the ‘fourth corner problem’ (Brown et al. 2014). Generalised linear models (GLMs) were fitted to the matrix of species’ abundances (L) as a function of the cover of habitat morpho-functional groups (R), species traits (Q) and their interaction (R*Q), via the ‘mvabund’ R package (Wang et al. 2012). Models were implemented with a negative binomial distribution for over-dispersed count data. Model selection was done using a least absolute shrinkage and selection operator (LASSO penalty), which automatically sets to zero any interaction that does not improve the model fit. The significance of the ‘trait*environment’ (R*Q) interaction was calculated using 1000 resampling iterations by probability integral transform (PIT-trap) block resampling to account for correlation in testing. Prior to analyses, categorical traits were converted to dummy variables. Assumptions of linearity and heteroscedasticity were visually inspected by plotting residuals *vs.* fitted values (Fig. S2).

Second, we performed a RLQ analysis to explore the shared co-structure between the environmental (R), species’ abundance (L), and trait (Q) matrices. The RLQ analysis is a multivariate ordination method that allows testing multiple traits vs. multiple environments, rather than the pairwise testing of the ‘fourth-corner’ method. This method combines a correspondence analysis on the L matrix and principal component analyses on the Q and R matrices, using the scores of the sampling sites and species from the previous correspondence analysis on matrix L as weight of the rows (Dolédec et al. 1996; Dray et al. 2014). The RLQ axes quantify the cross-covariance between environmental and trait ordinations. Variables with the highest positive or negative score on the RLQ axes contribute most to the observed spatial variation in species’ abundances and trait–environmental relationships, while variables with a score close to 0 have a negligible contribution. Analyses were carried out in the ‘ade4’ R package (Dray and Dufour 2007).

### Trophic and energetic dynamics

To investigate how potential habitat reconfigurations may influence energy storage and flow in reef fishes, we quantified standing biomass (scaled to kg ha^−1^) and two dynamic, flow-based, ecosystem-level metrics: secondary productivity (i.e. amount of biomass produced per day; scaled to kg ha^−1^ day^−1^) and turnover (i.e. the proportional flow of biomass in the system, either as being incorporated or release; % day^−1^) (Morais et al. 2020; Tebbett et al. 2021). To estimate secondary productivity, we used the individual age framework recently developed by Morais and Bellwood (2020), implemented in the ‘rfishprod’ R package. This framework uses information on individual fish body size (obtained from survey data), sea surface temperature (SST) for the study region, ageing method (otolith’s rings), and species-level trait information (maximum length, diet, and position in the water column) to predict k_max_—a standardized Von Bertalanffy growth coefficient representing the rate at which a fish would approach its asymptotic length—for unsampled species (Morais and Bellwood 2018). The SST was sourced from daily satellite measures obtained from the NOAA optimally interpolated data (Reynolds et al. 2007), at ~ 1 km spatial resolution and averaged across the sampling dates. Trait information was sourced from the dataset provided in Morais and Bellwood (2018) and other published sources (mainly Fishbase, https://www.fishbase.de/). Ageing method was set to a fixed level (i.e. otolith’s rings) in the Gradient Boosted Regression Trees models, as this information is not available for unsampled species, as the otolith method has been demonstrated to perform better than others (e.g. length-frequency) that tend to overestimate k_max_. The expected biomass gain after a day (i.e. somatic growth) was computed by placing each fish in its predicted growth trajectory (i.e. length increments), and then converted to biomass using published length–weight relationships for each species. We then applied a size- and species-specific mortality risk coefficient to simulate the fishes that would stochastically perish after a day, using the empirical relationships derived by Pauly (1980) and Gislason et al. (2010), which considers growth trajectories and sea surface temperature with an exponential negative relationship with individual-body size. We note these relationships do not account for varying levels of fishing mortality across species. The secondary productivity of each transect was then estimated by summing the somatic growth of surviving fishes after a day of growth, while biomass turnover was computed as the quotient of produced to standing biomass (Allen 1971).

To investigate shifts in the main trophic pathways that underpin energy transfer through the food web (Morais and Bellwood 2019), we calculated metrics of energy flow and storage both at the system-level (all species pooled), as well as for each major trophic guild in the dataset. For this, trophic guild levels used in analyses of community assembly (Table S2) were grouped into coarser categories representing main trophic interactions in reef fishes, and hence the pathways through which primary production is efficiently transferred to upper trophic levels (Parravicini et al. 2020). These included herbivores/detritivores, higher carnivores, microinvertivores, planktivores, and sessile invertivores. Browsing herbivores that consume the canopy-forming kelp and *Sargassum* spp. (mainly drummers, *Kyphosus* spp.) were only present in a limited number of transects, and were, therefore, excluded from the analyses.

We predicted and tested for differences in fish biomass, productivity and turnover among habitats (fixed factor), pooled and for each trophic guild, using GLMs, implemented in the ‘glmmTMB’ R package (Brooks et al. 2017). The GLMs were fitted using a Gamma error family structure with a log link function, which is suitable for continuous positive response variables, and a Tweedie error family structure with a log link function, which is suitable for right-skewed and overdispersed data (see Table S5 for details on each response metric), with *p* values obtained via a likelihood ratio test. All models were investigated for violations of statistical assumptions via quantile–quantile plots, test of zero-inflation, and residual *vs.* fitted values. Sessile invertivores had a large number of missing turnover values, and were excluded from this analysis. We additionally calculated the contribution (%) of each reef fish family and trophic guild to patterns of total abundance, biomass and productivity across habitats.

## Results

### Patterns of community assembly

**Fig. 2:**
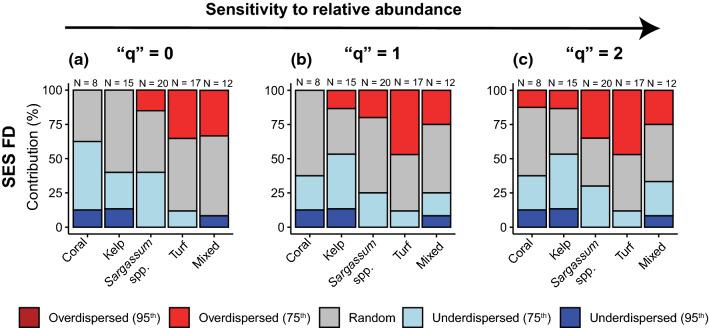
Relative contribution of over-dispersion (i.e., divergence, red), under-dispersion (i.e., convergence, blue), and randomness (grey) to patterns of reef fish community assembly across habitats. The pattern of standardized effect size functional diversity (SES FD) was investigated under increasing sensitivity to functional entities’ relative abundance: **a** “*q*” = 0 (species compositions only), **b** “q” = 1 (higher weight on common entities’), and **c** “*q*” = 2 (higher weight on dominant entities’). Dark red (over-dispersed) and blue (under-dispersed) colours depict assemblages that were significantly different from the null model (outside the 95th percentile of the null distribution). Sample sizes (*N*) for each habitat category are included above barplots

Coral and kelp habitats displayed a higher proportion of functionally convergent assemblages (i.e. under-dispersed), with ~ 15% of the transects containing species that were significantly (i.e. > 95th percentile of the null distribution) more functionally similar than expected by random chance, irrespective of the weight on functional entities’ relative abundances (Fig. 2). In contrast, *Sargassum* spp. and turf habitats contained a higher proportion of functionally divergent assemblages (i.e. over-dispersed, ~ 25% of transects), although the pattern was not statistically different from the null model (i.e. values between the 75th and 95th percentile of the null distribution), irrespective of the weight on relative abundances (Fig. 2). Mixed habitats displayed signals of both functional convergence (~ 10% of transects) and divergence (~ 30% of transects). Neutral processes also largely contributed to reef fish community assembly, independently of the habitat group and the weight on functional entities’ relative abundances.

### Fish trait–habitat relationships

**Fig. 3:**
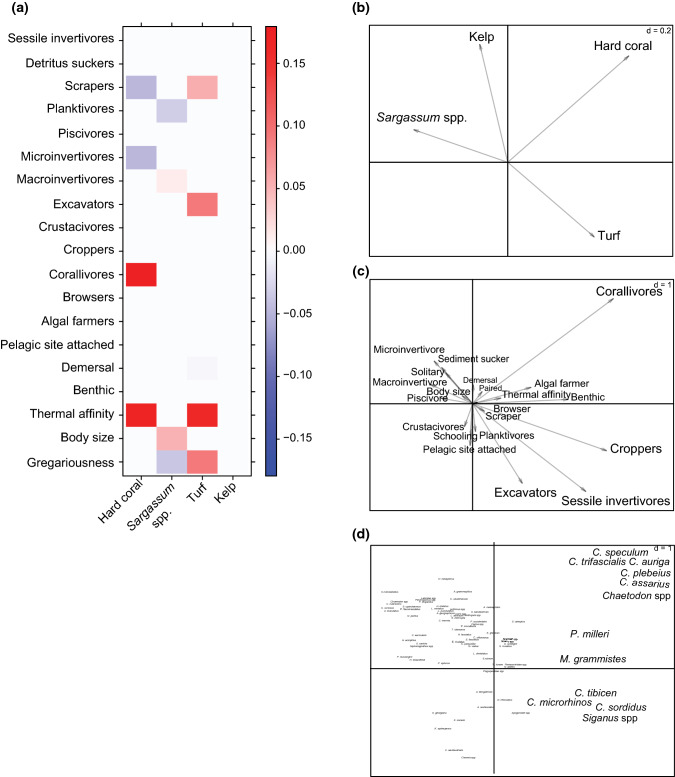
Trait–environmental relationships. **a** Fourth-corner analysis testing the interaction between functional traits and the percent cover (%) of habitat groups to predict the abundance of fish species. Colour intensity denotes the magnitude of the standardised coefficients (β) from GLMs with a LASSO penalty for model selection. Red = positive correlations, blue = negative correlations. **b**, **c** Shared co-structure between the percent cover of habitat groups (**b**), fish traits (**c**), and fish abundance (**d**) tables in RLQ analyses. In (**d**), only species with higher scores in the RLQ axes are magnified for visualization purposes. RLQ scores for trait and environmental variables are shown in Fig. S3, whilst species codes are provided in Table S6

The variation of fish species’ abundances across habitats was mediated by their functional traits (‘fourth-corner’ analysis, ‘environment*trait’, d*f* = 68, dev = 220.5, *p* = 0.01), although the effect (standardised coefficients, β) was relatively weak for most traits considered (Fig. 3a). Trophic guild was one of the most important traits, with specialised corallivorous fishes displaying the stronger, positive, association with coral habitats. The scraping parrotfishes displayed a negative association with coral habitats, whilst their abundances were positively correlated with increased cover of turf. Likewise, excavators displayed a positive association with turfs. Microinvertivorous fishes displayed a negative association with the percent cover of coral, while fishes exploiting planktonic sources displayed a negative association with the percent cover of *Sargassum* spp. Thermal affinity was also an important functional trait explaining variation in species’ abundances, with warm-affinity species being more abundant in coral and turf habitats. Although weaker in their relative effect, body size and gregariousness also contributed to explain variation in species’ abundances, with larger solitary species partly associated with *Sargassum* spp. habitats.

The RLQ analysis provided complementary support to pairwise relationships identified in the ‘fourth-corner’ method. The first (53.8%) and second (28.3%) RLQ axes cumulatively explained 82.1% of the cross-covariance between traits and environment across species and sampling units (Fig. S3). Similar to the ‘fourth-corner’ method, the traits best represented (highest positive or negative scores) by RLQ axes were related to resource use (trophic guild) and thermal affinity (Fig. S3). The best associations between trait and environment occurred for coral and turf habitats, with specialized corallivorous fishes of the family Chaetodontidae aligning closely with turf habitats in the ordination space, whilst croppers, sessile invertivores, and excavators aligned closely with turf habitats (Fig. 3b, c). Thermal affinity also aligned closely with the RLQ axis 1, which separated turf and coral habitats from canopy forming kelp and *Sargassum* spp. habitats, with characteristically warm-affinity species occupying this space of the ordination plot (Fig. 3d).

### Trophic and energetic dynamics

**Fig. 4:**
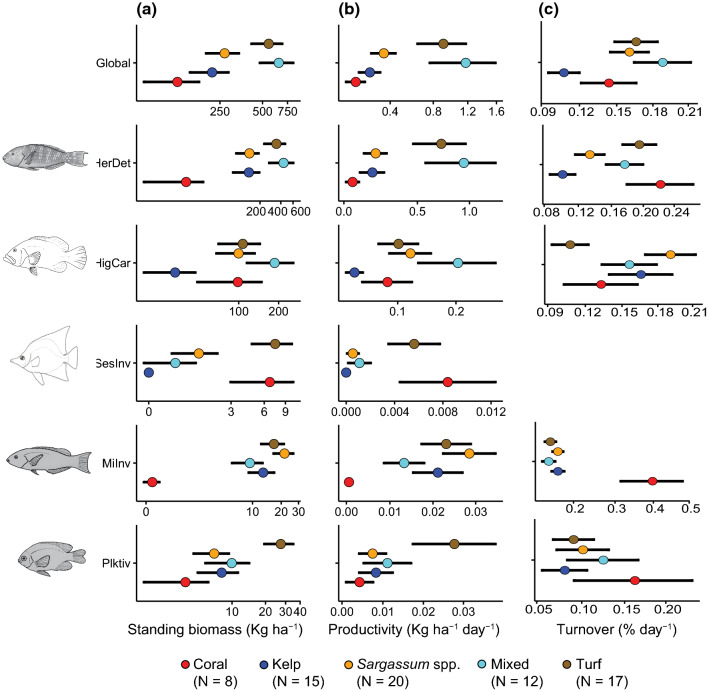
Predicted (mean ± SE) **a** standing biomass, **b** productivity, and **c** turnover of reef fish trophic guilds, from GLMs, across habitat groups: coral (red), kelp (blue), mixed (cyan), *Sargassum* spp. (orange), and turf (brown). *HerDet* herbivores/detritivores, *HigCar* higher carnivores, *SesInv* sessile invertivores, *MiInv* microinvertivores, *Plktiv* planktivores. For visualization purposes standing biomass is shown on a log scale. Sample sizes for each habitat category are included in the legend

The standing biomass, productivity and turnover of reef fish assemblages (pooled) significantly differed among habitats (Table S5). Mixed and turf habitats supported the highest averaged fish biomass, while *Sargassum* spp., kelp, and coral habitats supported lower, and statistically comparable, fish biomass (Fig. 4a). Patterns of fish productivity paralleled those of fish biomass, with remarkably higher productivity in mixed and turf habitats compared to that found in *Sargassum* spp., kelp, and coral habitats (Fig. 4b). Despite supporting lower fish biomass and productivity, *Sargassum* spp. and coral habitats displayed statistically comparable levels of averaged biomass turnover rates to that found on mixed and turf habitats, while kelp habitats supported the lowest averaged biomass turnover rates (Fig. 4c).

Among-habitat differences in fish biomass and productivity were contingent on the trophic guild (Table S5). Herbivores/detritivores largely contributed to the observed assemblage-level patterns (Fig. 4, Fig. S4), with mixed and turf habitats supporting on averaged ~ three times more biomass and ~ four times more productivity than *Sargassum* spp. and kelp habitats (Fig. 4a, b). Coral habitats supported the lowest biomass and productivity of herbivores/detritivores, with mixed and turf habitats supporting ~ 21 times more biomass and ~ 15 times more productivity. Higher carnivores displayed statistically comparable levels of biomass, but their productivity differed among habitats (Table S5). Mixed habitats supported the larger productivity of higher carnivores, followed by *Sargassum* spp., turf, and coral habitats, while kelp habitats supported the lowest productivity (Fig. 4b). Sessile invertivores had significantly higher biomass and productivity in coral and turf habitats, whilst this group had an almost negligible biomass and productivity in *Sargassum* spp., mixed, and kelp habitats (Table S5, Fig. 4a, b). The biomass and productivity of microinvertivorous fishes significantly differed among habitats (Table S5), being almost negligible in coral habitats and comparable among *Sargassum* spp., turf, kelp, and mixed habitats (Fig. 4a, b). Planktivorous fishes had significantly higher biomass in turf habitats, but their productivity was comparable among habitat types (Table S5, Fig. 4a, b).

While biomass and productivity generally unveiled similar among-habitat patterns, turnover generally displayed an inverted pattern (Fig. 4c, Table S5). This was largely driven by the high biomass turnover rates encountered in coral habitats for herbivores/detritivores, microinvertivores and planktivores, relative to their very low biomass and productivity in this habitat. This inverted pattern was partly related to the generally smaller sized individuals found in coral habitats for reef fish families contributing to these functions (Serranidae, Scaridae, Pomacentridae, Labridae, and Chaetodontidae) (Fig. S5), although there was high heterogeneity in the body size distribution of the speciose Labridae family in coral habitats.

## Discussion

Our study used a tropical–temperate biogeographic transition zone characterized by seascape-scale patches of coexisting reef-building corals (*Acropora* spp.), kelp forests (*Ecklonia radiata)*, *Sargassum spp.* beds, and turf to test the potential trophic and energetic consequences of predicted biogenic habitat reconfigurations under future warming scenarios (Vergés et al. 2019). We showed that reef fish species co-occurring in coral and kelp habitats displayed greater signals of trait convergence, highlighting a directional selection towards functional strategies that maximizes the use of the available niche space provided by these habitats (Winemiller et al. 2015). In contrast, species co-occurring in turf and *Sargassum* spp. habitats displayed greater signals of trait divergence, potentially signalling ecological opportunities minimizing niche overlap and thus enhancing species’ coexistence (Mcgill et al. 2006). The local filtering of species’ traits had an imprint on the trophic and energetic dynamics of reef fish assemblages, with remarkably high secondary productivity in turf and mixed habitats compared to *Sargassum* spp., kelp, and coral habitats. Despite these perceived gains in fish biomass production, turnover (i.e. the rate of biomass flow) was often decoupled for most trophic guilds, particularly for fishes that act as conduits of energy from primary producers to higher trophic levels (i.e. microinvertivores). Higher turnover rates in coral compared to turf habitats, despite their substantially lower productivity, have important implications for conservation and fisheries management, questioning the ability of structurally degraded reef configurations to maintain fish productivity over longer timescales (Robinson et al. 2019; Morais et al. 2020).

The higher proportion of functionally convergent assemblages found in coral and kelp habitats might be related to their higher physical structural complexity. This complexity can offer increased protection against predation to species with particular sizes (e.g. small-bodied) or behaviours (e.g. cryptic) (Graham and Nash 2013; Rogers et al. 2018a, b), while limiting the accessibility to nutritional resources to species with specialised diets (Beger, 2021; Stuart-Smith et al. 2021). For instance, in our study region, the abundance of highly specialised corallivorous fishes (family Chaetodontidae) displayed a positive association with the cover of reef-building corals, supporting previous observations in coral reefs (Pratchett et al. 2011), and highlighting their vulnerability to coral mortality (Graham et al. 2011), irrespective of the thermal environment (Stuart-Smith et al. 2018). Similarly, many temperate reef fish species in the region are habitat specialists, dwelling within vegetated habitats to minimize predation risk (Tuya et al. 2009). Habitat selection by specialist species can thus mediate the range-expansion success of tropical fishes into temperate regions (Beck et al. 2017), as exemplified by the close association between warm-affinity species and the cover of hard coral and turf habitats. This highlights the importance of local biotic factors in predicting climate-driven species redistributions, questioning model approaches that solely rely on regional climatic trends (Fernandes et al. 2020).

A plausible mechanism explaining the differences in thermal affinity found between canopy (kelp and *Sargassum* spp.) and non-canopy (turf) habitats might be related to the availability of nutritional resources exploited by herbivores. This is particularly true for those functional groups exploiting the resources contained within turfs (e.g. detritus, algae, and cyanobacteria), as these can become more available following the loss of foundation species and expansion of algal turfs (Gilmour et al. 2013; Pessarrodona et al. 2021a, b, c). The higher turnover rates (Bonaldo and Bellwood 2011) and lower chemical and physical defences (Littler et al. 1983) of the algae within turfs might further enhance the ecological opportunities provided by this habitat, compared to canopy-forming species, such as kelps, that are generally consumed by a few specialised families (e.g. Kyphosidae, Knudsen et al. 2019). In contrast, the resources contained within turfs are typically exploited by a diverse suite of tropical herbivorous fishes (e.g. algal farmers, croppers, detritus suckers, scrapers) (Vergés et al. 2014), which have evolved morphological and behavioural adaptations to minimize competition for resources across spatial and temporal axes (Siqueira et al. 2019).

An emerging question in the Anthropocene is how the loss of foundational species and their replacement by structurally simplified habitats (turf here) or alternative foundation species (*Sargassum* spp. here) might shifts the trophic and energetic pathways that underpin ecosystem functions and the delivery of food and other services to human societies (Bellwood et al. 2018; Vergés et al. 2019). Our results indicate that some pathways, particularly those that rely on the availability of basal tropic resources (i.e. herbivores/detritivores), might be enhanced, at least, over short timescales. This effect was particularly strong in the case of scraping parrotfishes (Scaridae), which disproportionately accounted for the abundance, biomass, and productivity of herbivores/detritivores in turf habitats (Fig. S6). These fishes are highly specialized in digesting and assimilating protein-rich autotrophic microorganisms (Clements et al. 2017), that can rapidly grow on dead coral or bare reef following disturbances (Diaz-Pulido and McCook 2002). In our study region, turfs supported remarkably high productivity of herbivores/detritivores (0.71 ± 0.24 kg ha^−1^ day^−1^). Of this, ~ 98% (0.70 kg ha^−1^ day^−1^) was supported by scraping parrotfishes, attaining even higher productivity to that reported for some tropical reefs that have been severely disrupted by environmental disturbances and the rise of turfs (~ 0.40 kg ha-1 day-1, Morais et al. 2020). Together with recent evidence from coral reef ecosystems globally (Taylor et al. 2019), these results point towards a bottom-up control of herbivores exploiting turf-driven pathways, challenging their classic view as agents of reef resilience through top-down control on benthic algae (Russ et al. 2015). Parrotfishes, thus, appear as climate winners over the short-term, albeit local scale factors such as sedimentation could offset the nutritional benefits provided by increased turf availability (Tebbett et al. 2021; Pessarrodona et al. 2021a, b, c).

Microinvertivorous fishes, another trophic guild that act as an important conduit of energy to higher trophic levels (Taylor 1998), displayed relatively comparable biomass and productivity in turf, mixed, *Sargassum* spp., and kelp habitats, while these metrics were almost negligible in coral habitats. The structural complexity of fucoids (e.g. *Sargassum* spp.) and laminariales (e.g. the kelp *Ecklonia radiata*), provide microhabitat refugees to small motile invertebrates living as epifauna (e.g. amphipods, isopods) (Edgar 1983). Although, in theory, the flattening of reef structural complexity should decrease the amount of microhabitat refugees provided to small motile invertebrates (Pessarrodona et al. 2021a, b, c), turfs growing on dead coral have been shown to maintain even higher epifaunal productivity than healthy live coral (e.g. Taylor 1998; Fraser et al. 2021). This high epifaunal productivity in turf habitats can be underpinned by bottom-up (e.g. higher availability of detrital and algal sources) and top-down (i.e. scale-dependence changes in the availability of microhabitat refugees) mechanisms (Fraser et al. 2021). The latter is reflected in the epifaunal composition of each habitat, with turfs being majorly composed of small-sized taxa with high turnover rates (mainly harpacticoid copepods) (Fraser et al. 2020b). In contrast, colonization of corals by smaller sized epifauna can be deterred by chemical and physical defence strategies (Sammarco et al. 1983), as well as potential heterotrophy of coral polyps (Goreau et al. 1971), with larger, slower paced, taxa (mainly decapods) inhabiting this habitat (Fraser et al. 2020b).

Despite the overall gains in fish biomass and productivity in turf and mixed habitats, turnover (i.e. the rate of biomass flow) generally displayed contrasting patterns. Part of these among-habitat differences might stem from the different thermal affinity of the composite assemblage, as tropical species typically have faster growth rates, early maturation, and shorter lifespans than temperate ones (Beukhof et al. 2019). However, for some trophic guilds, turf and coral habitats displayed markedly contrasting patterns, despite both containing a higher proportion of tropical species. For instance, microinvertivores displayed remarkably higher turnover rates in coral habitats, despite their biomass and productivity being substantially larger in turf. The higher productivity, at the expense of lower turnover, indicates potential biomass storage effects in turf habitats for these trophic guilds, whereby a potential short-term increase in the accessibility to preys due to degraded reef structural complexity can drive energetic shifts towards biomass accumulation (Morais et al. 2020). In contrast, the complex branching structure of *Acropora* spp. corals can provide increased protection against predation for early life history stages (Rogers et al. 2018a, b), enhancing their survival and replenishing stock biomass as adult fishes perish (Rogers et al. 2018a, b). This was reflected in our study by the generally smaller size of fishes found in coral habitats for the family Labridae, whose constituent species are major contributors to the microinvertivorous trophic pathways (Fig. S5).

Our study presents a number of caveats that warrant caution when transferring these results to other reef systems. First, it must be noted that habitats at the study area were sometimes interspersed within scales of 100 s to 1000 m (i.e. at the site-scale). Thus, it is plausible that transects with dominance of one habitat type, still recorded a proportion of species inhabiting nearby habitats due to the generally large home range sizes and mobility of adult demersal fishes (Nash et al. 2015). This limitation also entails pseudo-replication among some habitat level comparisons, potentially biasing parameter estimates and inferences of statistical significance (Davies and Gray 2015), particularly for those trophic guilds that displayed marginally significant or non-significant results. Finally, as any sampling method, stereo-DOVs are subjected to varying detectability of fish species among habitats (Holmes et al. 2013). For instance, small-bodied species with low mobility and cryptic behaviour could have been undersampled in vegetated habitats (kelp and *Sargassum* spp.) (French et al. 2021), as well as in coral habitats due to the complex morphology of the staghorn *Acropora* spp. corals, compared to turf habitats. This methodological bias could have potentially influenced the taxonomic and functional characterization of fish assemblages (Esmaeili et al. 2021), and hence the degree to which we were able to detect patterns of functional divergence and convergence in each habitat. We must note then, that the patterns reported here apply at least to mobile conspicuous species, which are generally observed moving both within and above fronds of vegetated habitats (kelp and *Sargassum* spp.), as well as the complex branching structure of *Acropora* spp. corals. Future studies should use a combination of methods to capture both conspicuous and cryptic species to discern the generality of the assembly rules reported here, particularly considering the fine-scale partitioning of available micro-niches by cryptobenthic fish species (Brandl et al. 2018).

Our study provides valuable insights on the deterministic processes involved in the assembly of fish communities across varying habitat configurations in a temperate-tropical transition zone, and signal potential predictable shifts in the delivery of key ecosystem functions (standing stock biomass, productivity, and turnover) that mediate reef health and the provision of food to human societies. Future studies should seek to decouple the direct and indirect links and effects between changes in environment, habitat, and aspects of biodiversity that scale-up to shape these three important ecosystem functions in rapidly changing tropical-temperate biogeographic transition zones. This is particularly important in the Anthropocene era, where increased frequency and intensity of climate disturbance events are likely to homogenise seascapes over regional and global scales (Dietzel et al. 2021; Pessarrodona et al. 2021a, b, c), disrupting the delivery of key ecosystem functions (Duffy et al. 2016; Maureaud et al. 2019). Thus, incorporating trends in local habitats changes with regional changes in ocean climate (e.g. temperature and productivity) appears critical to improve climate predictions on biodiversity-ecosystem functioning relationships (Fernandes et al. 2020). Given the broad applicability of functional traits, this will help to identify a range of winners and losers beyond biogeographic boundaries, and adapt management and conservation efforts that maximize the delivery of services to human societies whilst minimizing biodiversity loss.

## Supplementary Information

Below is the link to the electronic supplementary material.

Supplementary file1 (DOCX 1439 KB)

## Data Availability

The data and R code used for analyses can be made available upon request from the editor’s and reviewers through the author personal GitHub repository (https://github.com/NestorBosch).
