# Supplementary material for: Habitat configurations shape the trophic and energetic dynamics of reef fishes in a tropical–temperate transition zone: implications under a warming future
Source: Oecologia. 2022 Nov 7;200(3-4):455–70. doi: 10.1007/s00442-022-05278-6 (PMC9675646; doi:10.1007/s00442-022-05278-6)
Supplement: Supplementary file 1 — Supplementary file1 (DOCX 1439 KB) [file 442_2022_5278_MOESM1_ESM.docx]

# **Habitat configurations shape the trophic and energetic dynamics of reef fishes in a tropical-temperate transition zone: implications under a warming future**

Nestor E. Bosch^1^*, Albert Pessarrodona^1^, Karen Filbee-Dexter^1, 2^, Fernando Tuya^4^, Yannick Mulders^1^, Sahira Bell^1^, Tim Langlois^1^, Thomas Wernberg^1, 2, 3^

^1^ The UWA Oceans Institute, School of Biological Sciences, The University of Western Australia, 35 Stirling Highway, Crawley 6009, Western Australia, Australia

^2^ Institute of Marine Research, Nye Flødevigveien 20, 4817, His, Norway

^3^Department of Science and Environment, Roskilde University, 4000 Roskilde, Denmark

^4^ Grupo en Biodiversidad y Conservación, IU-ECOAQUA, Universidad de Las Palmas de Gran Canaria, Crta. Taliarte s/n, 35214, Telde, Spain

* Corresponding author: nbosch1989@gmail.com

**Keywords:** community assembly, ecosystem functions, ecosystem services, trait-based ecology, tropicalisation.

**Supplementary Materials**

**
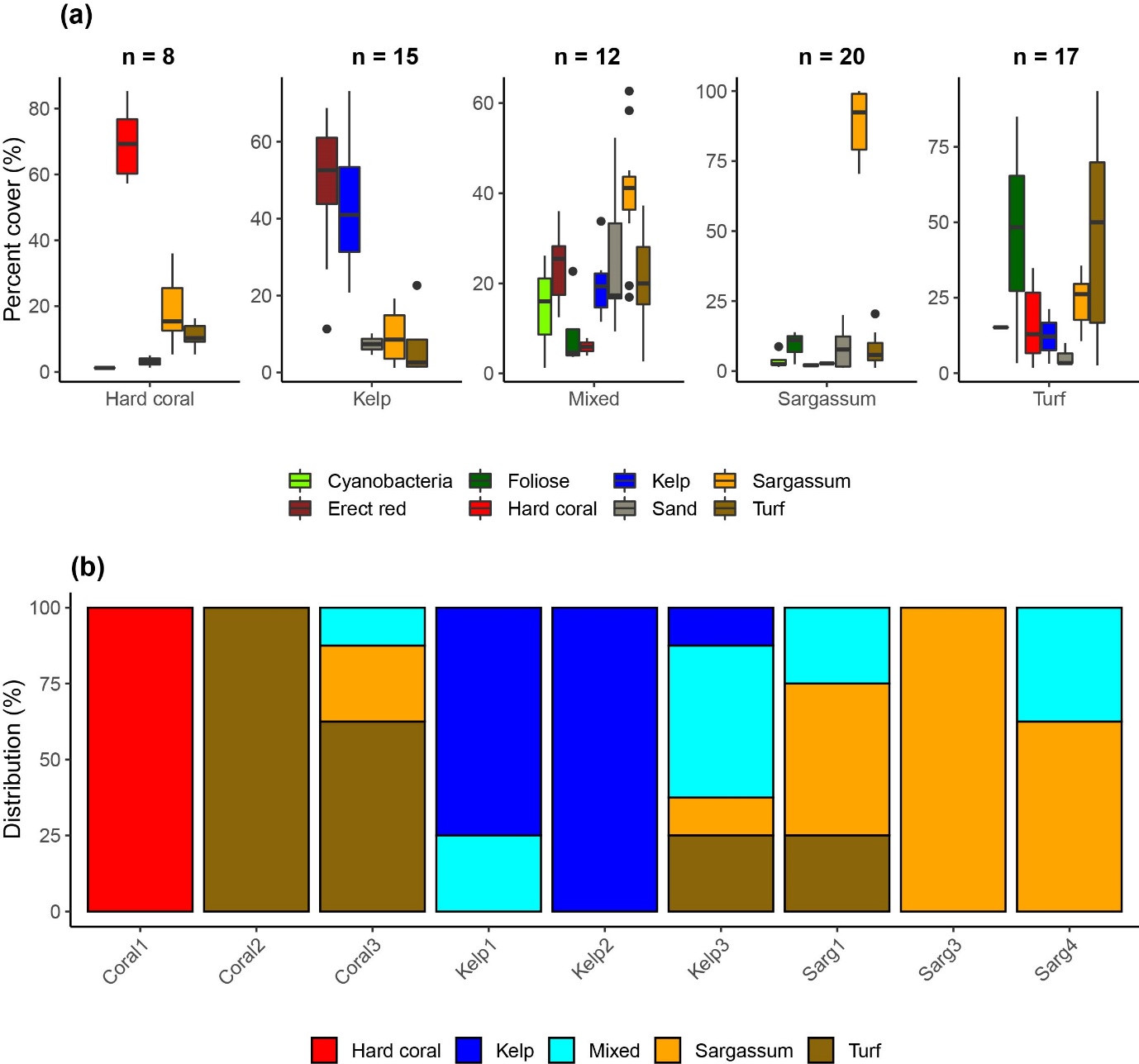
**

**Fig. S1. (a)** Boxplot distributions of percent cover (%) for habitat morpho-functional groups within each habitat cluster identified in the stereo-DOVs. The number of samples (n) at each habitat group is included within each panel. Black dots depict outlying values (i.e. < or > 1.5 x the inter-quartile range). **(b)** Distribution (%) of habitat clusters within each survey site


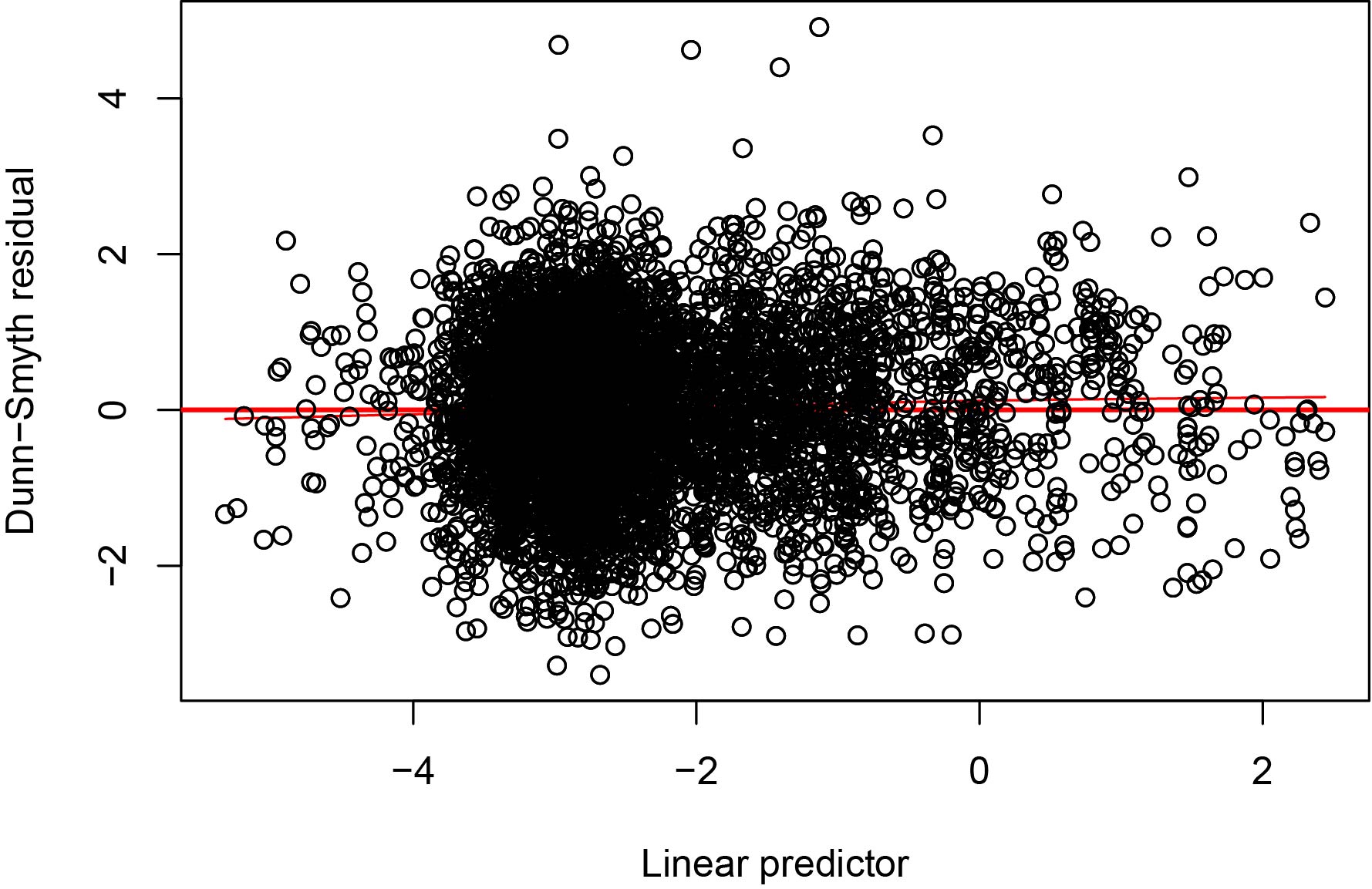


**Fig. S2.** Residuals *vs.* fitted values from generalised linear models testing the effect of habitat, species traits, and their interaction on the abundance’s of reef fish species


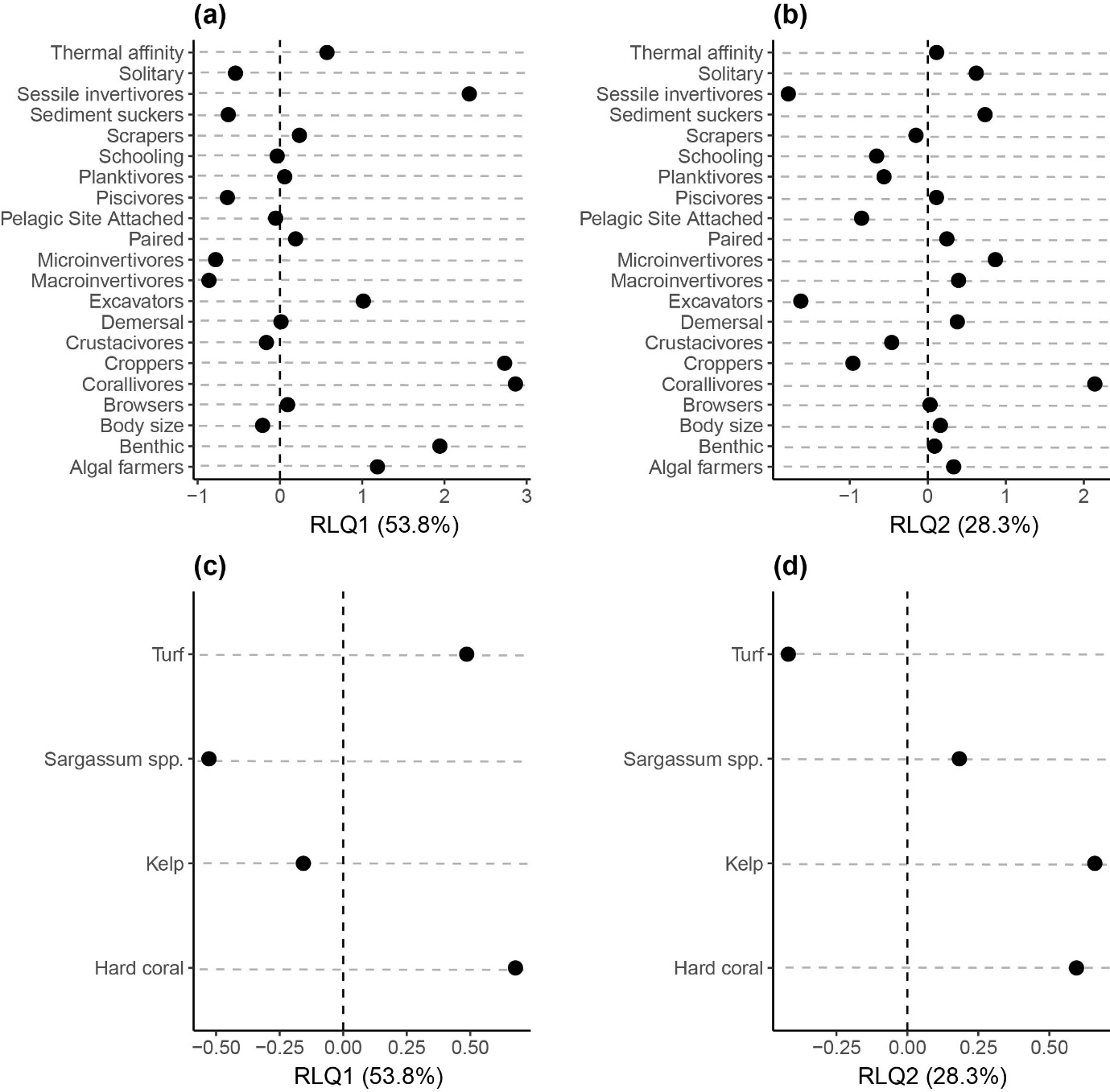


**Fig. S3.** Scores of the traits (a, b) and environmental variables (c, d) on RLQ axis 1 (a, c) and RLQ axis 2 (b, d). The percentage explained of the cross-covariance between traits and environmental variables across species and sampling units for each RLQ axis is indicated


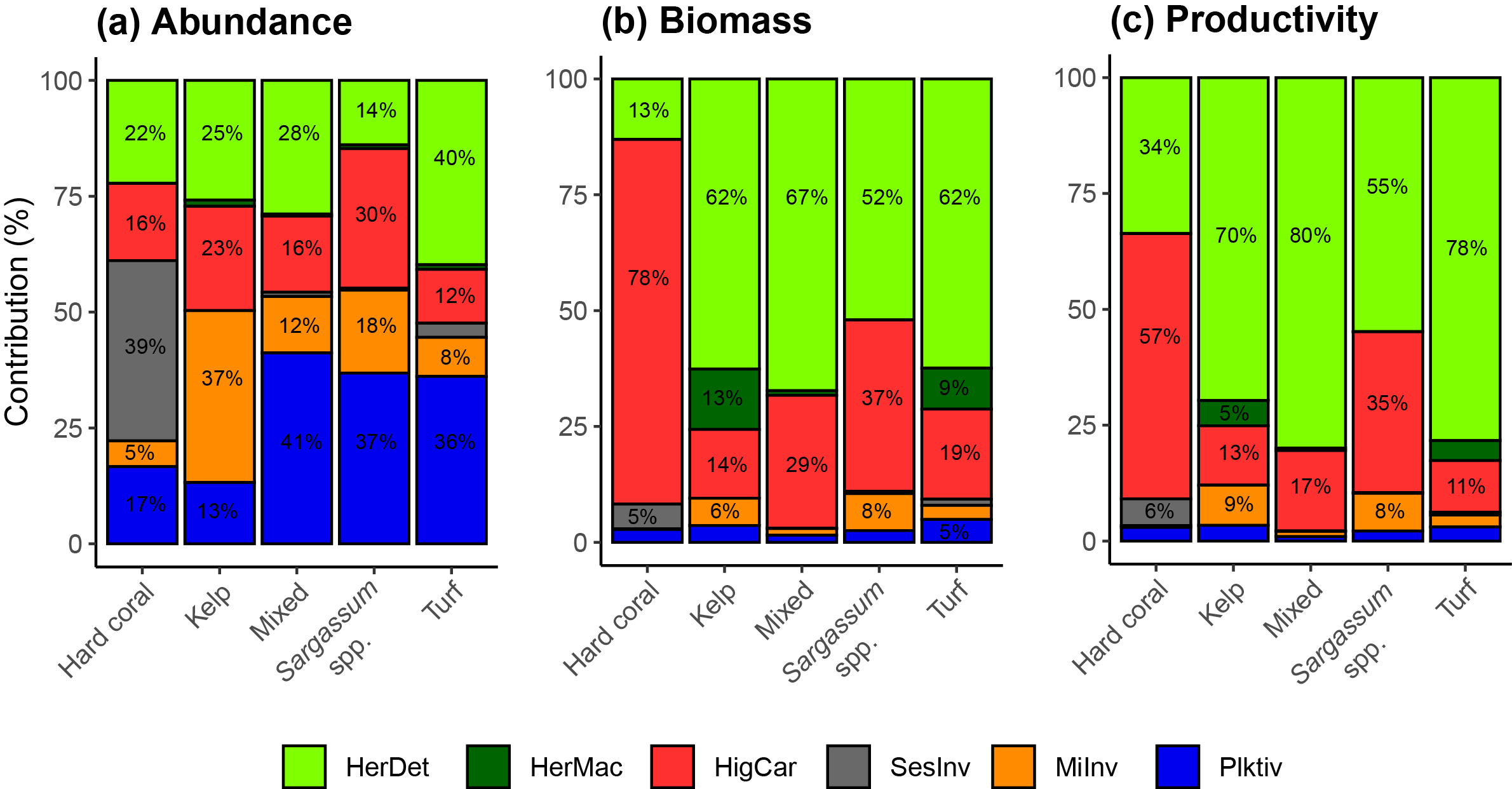


**Fig. S4.** Contribution (%) of each trophic guild to patterns of total fish (a) abundance (ind.125 m^-2^), (b) biomass (kg ha^-1^), and (c) productivity (kg ha^-1^ day^-1^) across habitat groups. For trophic guilds with a contribution ≥ 5%, the value is included within each stack bar. HerDet: herbivores/detritivores; HerMac: herbivores/browsers; HigCar: higher carnivores; SesInv: sessile invertivores; MiInv: microinvertivores; Plktiv: planktivores


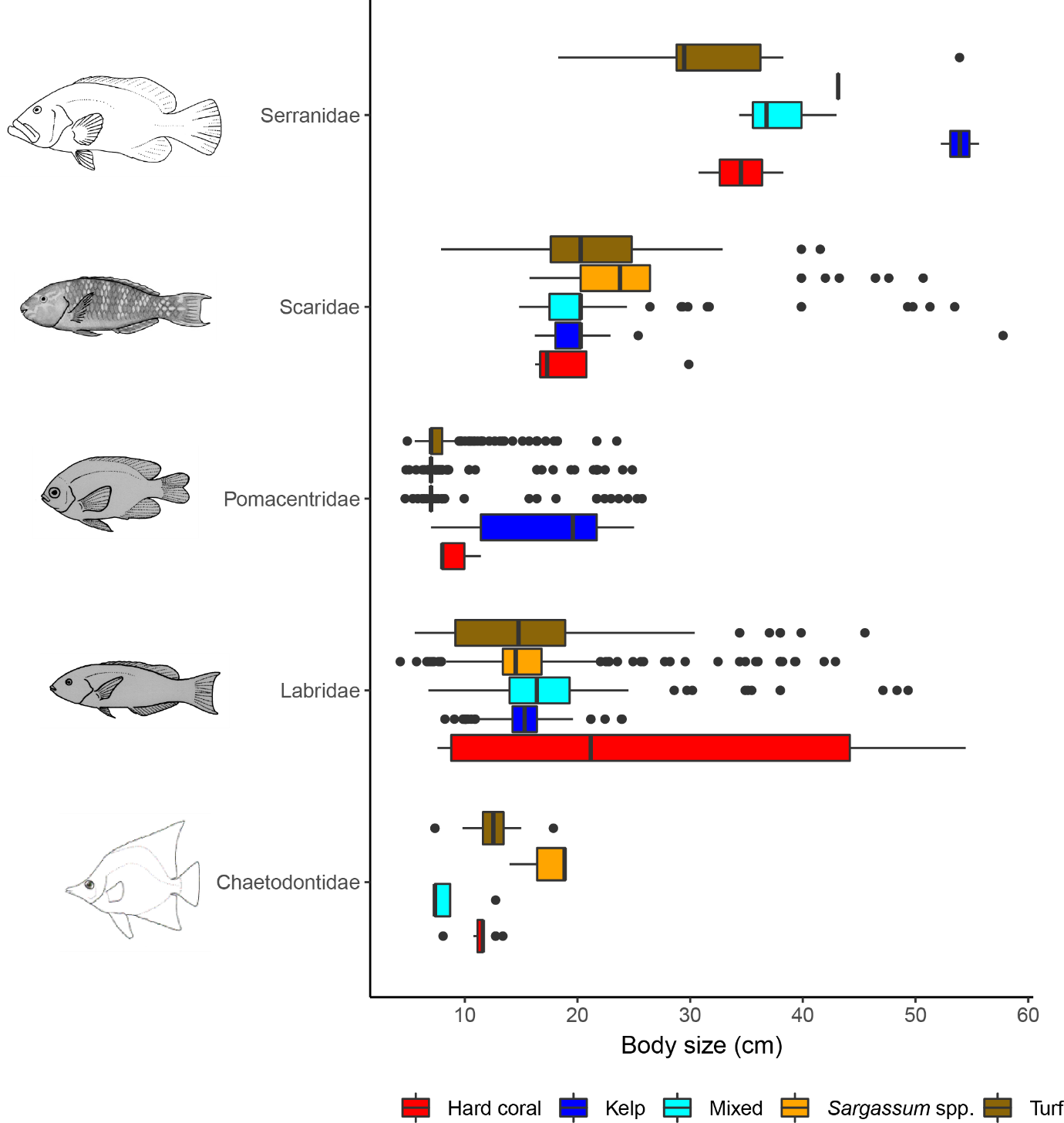


**Fig. S5.** Boxplot distributions of body size (cm) for the main reef fish families across habitat groups: coral (red), kelp (blue), mixed (cyan), *Sargassum* spp. (orange), and turf (brown). Black dots depict outlying values (i.e. < or > 1.5 x the inter-quartile range)


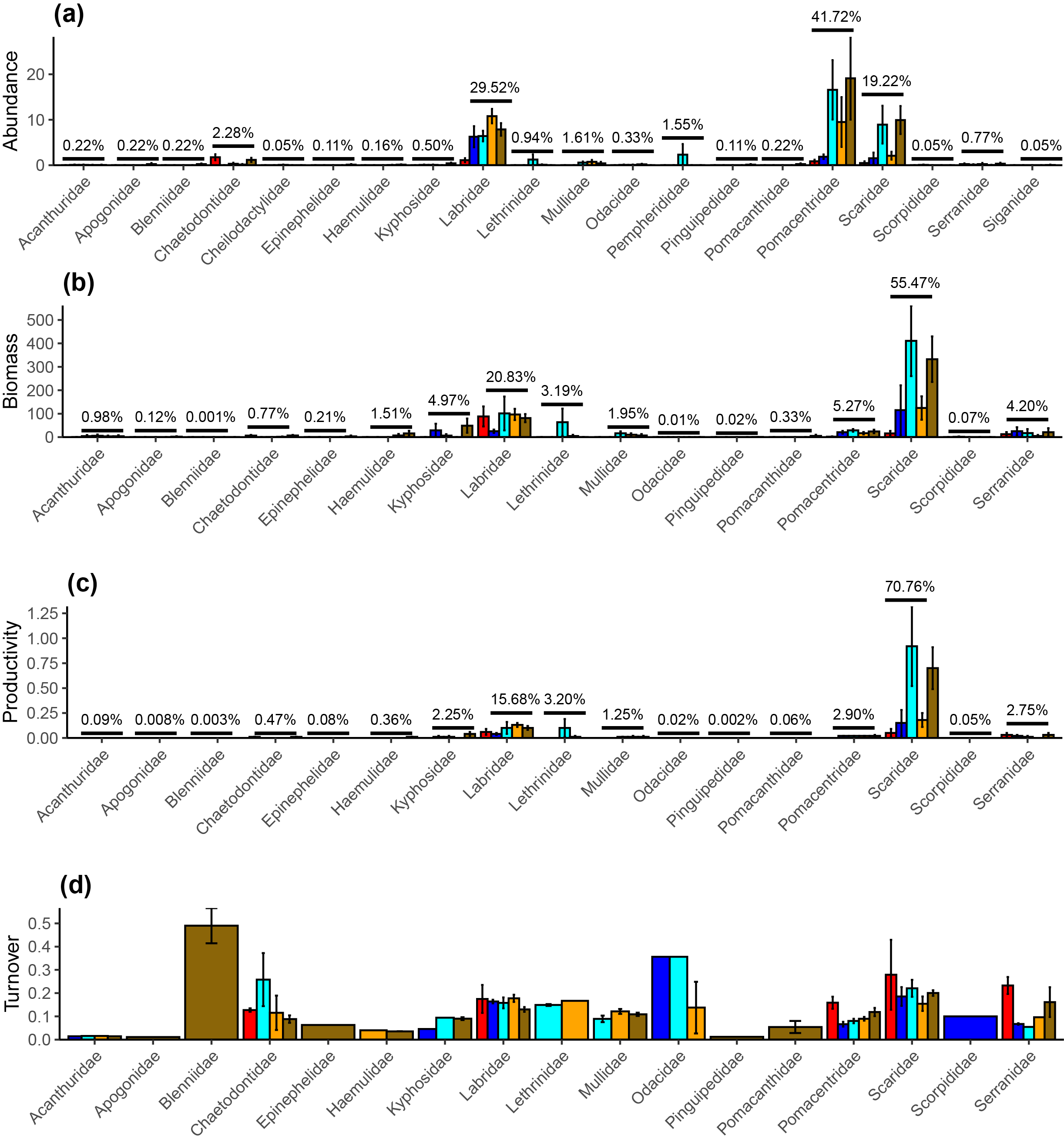


**Fig. S6.** Mean (± SE) abundance (ind.125 m^-2^), biomass (kg ha^-1^), productivity (kg ha^-1^ day^-1^), and turnover (% day^-1^) across habitat groups, for each reef fish family. The contribution (%) to total fish abundance, biomass, and productivity of each reef fish family is included within each panel

**Table S1.** Distribution of sampling effort (# transects) for fished and no-take areas at the study area.

|  | **Fished** | **No-take** |
| --- | --- | --- |
| **Hard coral** | 0 | 8 |
| **Kelp** | 14 | 1 |
| **Mixed** | 4 | 8 |
| ***Sargassum* spp.** | 12 | 8 |
| **Turf** | 10 | 17 |

**Table S2.** List of reef fish traits used in the analyses of community assembly rules across habitat groups.

| **Ecological traits** | **Type** | **Categories** | **Details** |
| --- | --- | --- | --- |
| Maximum length | Numeric | Maximum reported length obtained from Fishbase^1^ |  |
| Trophic guild | Factor | Planktivore^2^ | Species feeding in phyto- and zoo-plankton in the water column |
|  |  | Sediment sucker^3, 4^ | Herbivorous guild composed of species suctioning detritus from the turf |
|  |  | Microinvertivore^2^ | Species feeding on smaller-sized invertebrates (e.g. Annelida, Peracarida) |
|  |  | Sessile invertivores^2^ | Species feeding on sessile invertebrates (mainly Asteroidea, Bryozoa, Porifera, etc.) |
|  |  | Corallivore^2^ | Species feeding on Anthozoa and Medusozoa |
|  |  | Crustacivore^2^ | Species feeding on large Crustacea (Decapoda and Stomatopoda) |
|  |  | Piscivore^2^ | Species feeding on Actinopterygii and Cephalopoda |
|  |  | Excavator^3, 4^ | Herbivorous guild removing chunks of the reef matrix whilst targetting cyanobacteria |
|  |  | Macroinvertivore^2^ | Species feeding on Mollusca, Echinoidea, and Ophiuroidea |
|  |  | Browser^3, 4^ | Herbivorous guild feeding on blades and thallum of macroalgae |
|  |  | Algal farmer^3, 4^ | Herbivorous guild farming diverse and productive stands of filamentous algae within small, defended, territories |
|  |  | Scraper^3, 4^ | Herbivorous guild removing turf algae and small portions of the reef matrix whilst targeting cyanobacteria |
|  |  | Cropper^3, 4^ | Herbivorous guild cropping filamentous algae within the turf |
| Gregariousness | Ordered factor | Singleton^5, 6^ | Species generally found as solitary adults |
|  |  | Paired^5, 6^ | Species found in pairs or forming small schools (< 20 ind.) |
|  |  | Schooling^5, 6^ | Species found in large schools (> 20 ind.) |
| Water column position | Factor | Benthic^5, 6^ | Sedentary species, living in close association with the bottom |
|  |  | Demersal^5, 6^ | Mobile species that live near the bottom |
|  |  | Pelagic non-site attached^5, 6^ | Species swimming in the water column, with large mobility (between reefs) |
|  |  | Pelagic site attached^5, 6^ | Swimming in the water column within a reef |
| Thermal affinity | Numeric | Midpoint between the 5th and 95th percentile of each species’ realised thermal niche^7^ |  |

**Table S3.** Mean (± SE) taxonomic (TD) and functional (FD) diversity values for each habitat type included in the analysis of community assembly rules, under varying “q” parameter (i.e. sensitivity to species’ relative abundances): “q” = 0 (i.e., species composition only, analogous to species or functional richness), “q” = 1 (i.e., higher weight on common species, analogous to Shannon entropy for TD and divergence for FD), and “q” = 2 (i.e., higher weight on dominant species, analogous to inverse Simpson for TD and divergence for FD).

|  | **TD** | | | **FD** | | |
| --- | --- | --- | --- | --- | --- | --- |
| **Habitat** | **"q" = 0** | **"q" = 1** | **"q" = 2** | **"q" = 0** | **"q" = 1** | **"q" = 2** |
| Coral | 3.25 ± 0.49 | 3.04 ± 0.44 | 2.87 ± 0.40 | 2.08 ± 0.26 | 1.91 ± 0.21 | 1.85 ± 0.20 |
| Kelp | 3.80 ± 0.48 | 3.03 ± 0.40 | 2.75 ± 0.39 | 2.30 ± 0.23 | 1.99 ± 0.21 | 1.90 ± 0.21 |
| *Sargassum* spp. | 6.15 ± 0.85 | 4.70 ± 0.66 | 3.99 ± 0.55 | 2.68 ± 0.20 | 2.33 ± 0.18 | 2.22 ± 0.18 |
| Turf | 9.05 ± 1.23 | 5.54 ± 0.57 | 4.14 ± 0.39 | 3.66 ± 0.32 | 2.75 ± 0.20 | 2.48 ± 0.18 |
| Mixed | 7.41 ± 1.09 | 4.48 ± 0.78 | 3.57 ± 0.65 | 3.29 ± 0.34 | 2.35 ± 0.24 | 2.13 ± 0.23 |

**Table S4.** Correlation between Gower distance matrices using all traits and dropping one trait level each time for the analysis. Column headings refer to the trait drop each time for calculating Gower distances.

|  | **Gregariousness** | **Maximum length** | **Water column position** | **Thermal affinity** | **Trophic guild** |
| --- | --- | --- | --- | --- | --- |
| **All_traits** | 0.91 | 0.96 | 0.87 | 0.95 | 0.91 |

**Table S5.** Summary of GLMs testing for differences in standing biomass (kg ha^-1^), productivity (kg ha^-1^ day^-1^), and turnover (% day^-1^) across habitat groups for each reef fish trophic guild. The degrees of freedom (df), family error distribution and link function, likelihood ratio test (LRT), and p-values are indicated. Significant results (α < 0.05) are highlighted.

|  | **Standing biomass** | | | | **Productivity** | | | | **Productivity** | | | |
| --- | --- | --- | --- | --- | --- | --- | --- | --- | --- | --- | --- | --- |
|  | **Family (link)** | **df** | **LRT** | **p** | **Family (link)** | **df** | **LRT** | **p** | **Family (link)** | **df** | **LRT** | **p** |
| Global | Gamma ("log") | 4 | 12.82 | **0.01*** | Gamma ("log") | 4 | 21.29 | **<0.001*** | Gamma ("log") | 4 | 11.51 | **0.02*** |
| Herbivores/Detritivores | Tweedie ("log") | 4 | 15.7 | **0.003*** | Tweedie ("log") | 4 | 18.68 | **<0.001*** | Gamma ("log") | 4 | 15.22 | **0.004*** |
| Higher carnivores | Tweedie ("log") | 4 | 8.43 | 0.07 | Tweedie ("log") | 4 | 11.64 | **0.02*** | Gamma ("log") | 4 | 9.91 | **0.04*** |
| Sessile invertivores | Tweedie ("log") | 4 | 18.31 | **0.001*** | Tweedie ("log") | 4 | 20.45 | **<0.001*** |  |  |  |  |
| Microinvertivores | Tweedie ("log") | 4 | 22.54 | **<0.001*** | Tweedie ("log") | 4 | 19.9 | **<0.001*** | Gamma ("log") | 4 | 22.47 | **<0.001*** |
| Planktivores | Tweedie ("log") | 4 | 13.64 | **0.008*** | Tweedie ("log") | 4 | 8.22 | 0.08 | Gamma ("log") | 4 | 2.36 | 0.66 |

**Table S6.**

| **Species** | **Code** | **Species** | **Code** |
| --- | --- | --- | --- |
| *Chaetodon speculum* | *C. speculum* | *Anampses meleagrides* | *A. meleagrides* |
| *Chaetodon auriga* | *C. auriga* | *Acanthurus grammoptilus* | *A. grammoptilus* |
| *Chaetodon trifascialis* | *C. trifascialis* | *Coris caudimacula* | *C. caudimacula* |
| *Chaetodon plebeius* | *C. plebeius* | *Hologymnosus doliatus* | *H. doliatus* |
| *Chaetodon assarius* | *C. assarius* | *Lethrinus miniatus* | *L. miniatus* |
| *Chaetodon* spp | *Chaetodon* spp | *Labridae* spp | *Labridae* spp |
| *Siganus* spp | *Siganus* spp | *Plectropomus* spp | *Plectropomus* spp |
| *Chromis westaustralis* | *C. westaustralis* | *Plectropomus leopardus* | *P. leopardus* |
| *Chromis* spp | *Chromis* spp | *Choerodon* spp | *Choerodon* spp |
| *Apogonidae* spp | *Apogonidae* spp | *Choerodon rubescens* | *C. rubescens* |
| *Chlorurus microrhinos* | *C. microrhinos* | *Suezichthys cyanolaemus* | *S. cyanolaemus* |
| *Centropyge tibicen* | *C. tibicen* | *Plectorhinchus flavomaculatus* | *P. flavomaculatus* |
| *Chlorurus sordidus* | *C. sordidus* | *Austrolabrus maculatus* | *A. maculatus* |
| *Abudefduf bengalensis* | *A. bengalensis* | *Coris auricularis* | *C. auricularis* |
| *Dascyllus reticulatus* | *D. reticulatus* | *Heteroscarus acroptilus* | *H. acroptilus* |
| *Abudefduf sexfasciatus* | *A. sexfasciatus* | *Siphonognathus caninis* | *S. caninis* |
| *Scorpis georgiana* | *S. georgiana* | *Siphonognathus* spp | *Siphonognathus* spp |
| *Kyphosus cornelii* | *K. cornelii* | *Notolabrus parilus* | *N. parilus* |
| *Kyphosus sydneyanus* | *K. sydneyanus* | *Cheilio inermis* | *C. inermis* |
| *Hemigymnus melapterus* | *H. melapterus* | *Thalassoma lutescens* | *T. lutescens* |
| *Cheilodactylus rubrolabiatus* | *C. rubrolabiatus* | *Hemigymnus fasciatus* | *H. fasciatus* |
| *Pomacentrus milleri* | *P. milleri* | *Cheilinus chlorourus* | *C. chlorourus* |
| *Meiacanthus grammistes* | *M. grammistes* | *Epinephelus fasciatus* | *E. fasciatus* |
| *Parupeneus spilurus* | *P. spilurus* | *Gomphosus varius* | *G. varius* |
| *Halichoeres brownfieldi* | *H. brownfieldi* | *Chaetodontoplus personifer* | *C. personifer* |
| *Pempheris klunzingeri* | *P. klunzingeri* | *Epinephelus rivulatus* | *E. rivulatus* |
| *Pinguipedidae* spp | *Pinguipedidae* spp | *Parma* spp | *Parma* spp |
| *Sargocentron rubrum* | *S. rubrum* | *Parma occidentalis* | *P. occidentalis* |
| *Labroides dimidiatus* | *L. dimidiatus* | *Parma mccullochi* | *P. mccullochi* |
| *Scarus rivulatus* | *S. rivulatus* | *Stethojulis interrupta* | *S. interrupta* |
| *Scarus schlegeli* | *S. schlegeli* | *Stethojulis bandanensis* | *S. bandanensis* |
| *Scarus* spp | *Scarus* spp | *Stethojulis* spp | *Stethojulis* spp |
| *Scaridae* spp | *Scaridae* spp | *Lethrinus atkinsoni* | *L. atkinsoni* |
| *Stegastes obreptus* | *S. obreptus* | *Lethrinus* spp | *Lethrinus* spp |
| *Thalassoma lunare* | *T. lunare* | *Lethrinus punctulatus* | *L. punctulatus* |
| *Pomacentridae* spp | *Pomacentridae* spp | *Anampses geographicus* | *A. geographicus* |
| *Neopomacentrus aktites* | *N. aktites* | *Scarus ghobban* | *S. ghobban* |

**References**

^1^ Froese, R., & Pauly, D. (2021). FishBase.

^2^Parravicini, V., Casey, J. M., Schiettekatte, N. M., Brandl, S. J., Pozas-Schacre, C., Carlot, J., ... & Stuart-Smith, R. D. (2020). Delineating reef fish trophic guilds with global gut content data synthesis and phylogeny. PLoS biology, 18(12), e3000702.

^3^Bellwood, D. R., Streit, R. P., Brandl, S. J., & Tebbett, S. B. (2018). The meaning of the term ‘function’in ecology: A coral reef perspective. Functional Ecology, 33(6), 948-961.

^4^Siqueira, A. C., Bellwood, D. R., & Cowman, P. F. (2019). The evolution of traits and functions in herbivorous coral reef fishes through space and time. Proceedings of the Royal Society B, 286(1897), 20182672.

^5^ Stuart-Smith, R. D., Bates, A. E., Lefcheck, J. S., Duffy, J. E., Baker, S. C., Thomson, R. J., ... & Edgar, G. J. (2013). Integrating abundance and functional traits reveals new global hotspots of fish diversity. *Nature*, *501*(7468), 539-542.

^6^ Bosch, N. E., Gonçalves, J. M., Erzini, K., & Tuya, F. (2017). “How” and “what” matters: Sampling method affects biodiversity estimates of reef fishes. Ecology and Evolution, 7(13), 4891-4906.

^7^[Stuart-Smith, R. D., Edgar, G. J., Barrett, N. S., Kininmonth, S. J., & Bates, A. E. (2015). Thermal biases and vulnerability to warming in the world’s marine fauna. Nature. https://doi.org/](http://paperpile.com/b/VGpKln/6Xco)[10.1038/nature16144](http://dx.doi.org/10.1038/nature16144)
